# Vanillin Acrylate-Based Resins for Optical 3D Printing

**DOI:** 10.3390/polym12020397

**Published:** 2020-02-10

**Authors:** Aukse Navaruckiene, Edvinas Skliutas, Sigita Kasetaite, Sima Rekštytė, Vita Raudoniene, Danguole Bridziuviene, Mangirdas Malinauskas, Jolita Ostrauskaite

**Affiliations:** 1 Department of Polymer Chemistry and Technology, Kaunas University of Technology, Radvilenu Rd. 19, LT-50254 Kaunas, Lithuania; aukse.navaruckiene@ktu.lt (A.N.); sigita.kasetaite@ktu.lt (S.K.); 2 Laser Research Center, Faculty of Physics, Vilnius University, Sauletekis Ave. 10, LT-10223 Vilnius, Lithuania; edvinas.skliutas@ff.vu.lt (E.S.); sima.rekstyte@ff.vu.lt (S.R.); mangirdas.malinauskas@ff.vu.lt (M.M.); 3 Biodeterioration Research Laboratory, Nature Research Center, Akademijos Str. 2, LT-08412 Vilnius, Lithuania; vita.raudoniene@gamtc.lt (V.R.); danguole.bridziuviene@gamtc.lt (D.B.)

**Keywords:** vanillin dimethacrylate, vanillin diacrylate, photopolymerization, optical 3D printing, direct laser writing, replica molding

## Abstract

The investigation of biobased systems as photocurable resins for optical 3D printing has attracted great attention in recent years; therefore, novel vanillin acrylate-based resins were designed and investigated. Cross-linked polymers were prepared by radical photopolymerization of vanillin derivatives (vanillin dimethacrylate and vanillin diacrylate) using ethyl(2,4,6-trimethylbenzoyl)phenylphosphinate as photoinitiator. The changes of rheological properties were examined during the curing with ultraviolet/visible irradiation to detect the influences of solvent, photoinitiator, and vanillin derivative on cross-linking rate and network formation. Vanillin diacrylate-based polymers had higher values of yield of insoluble fraction, thermal stability, and better mechanical properties in comparison to vanillin dimethacrylate-based polymers. Moreover, the vanillin diacrylate polymer film showed a significant antimicrobial effect, only a bit weaker than that of chitosan film. Thermal and mechanical properties of vanillin acrylate-based polymers were comparable with those of commercial petroleum-derived materials used in optical 3D printing. Also, vanillin diacrylate proved to be well-suited for optical printing as was demonstrated by employing direct laser writing 3D lithography and microtransfer molding techniques.

## 1. Introduction

3D printing, also known as additive manufacturing, is a growing technology that has drawn increasing attention globally and has made a revolutionary impact on product fabrication in areas like medicine, the food industry, textiles, architecture, and construction [1]. Polymers are widely used in our everyday life due to their lightness, firmness, and relatively low cost; however, they are hard to form into intricate geometries. Additive manufacturing is the solution of creating complex geometries from plastics [2]. Several 3D printing technologies exist, allowing to create structures out of photo-curable resins. In stereolithography, resins are formed into 2D patterned layers [3], which become solid after exposing to UV light. By repeating this procedure, a 3D structure can be created layer by layer [4]. On the other hand, direct laser writing (DLW) 3D lithography can employ ultrashort pulses of focused VIS or near IR radiation instead of linearly absorbed UV light. This way a nonlinear light–matter interaction is achieved that confines the absorption and, subsequently, the cross-linking reaction in a small volume inside the resin, thus enabling full 3D (nonlayered) fabrication of structures with subwavelength feature sizes [5]. For 2D or quasi-3D structures, microtransfer molding (nanoimprint lithography) can be used. It employs the solidification of a resin (usually by UV light or thermally) inside the soft mold, thus replicating the master structure that was used to create the mold [6]. This technique is especially suitable in cases when many identical objects are required, as the same master structure can be used to make several molds, each suitable to create hundreds of replicas.

Advantages of photopolymerization are energy efficiency, high reaction rates, freedom from solvents [7], and the ability to photocure only the desired area of the resin [8]. Photopolymerization does not require any specific temperatures and can be performed at ambient conditions [9]. These features allow photopolymerization to be used in many engineering and manufacturing fields such as dental restoration, coatings, and 3D printing [10]. Radical photopolymerization is one of the most widely used photopolymerization processes. In this process, photoinitiator is used to generate radicals through cleavage and hydrogen abstraction reactions [11]. Acrylates and methacrylates are mostly used in free radical photopolymerization [12].

Vanillin is mostly obtained by extraction from the beans of vanilla orchids or by chemical modification of lignin, which is the second most prevalent biopolymer. Due to its aromatic structure, vanillin could replace widely used petro-based aromatic monomers [13]. Vanillin polymers exhibit re-processability and biodegradability under acid solution [14], as well as biodegradability in soil [15]. Recently, vanillin has been used to synthesize high-performance flame-retardant epoxy resins containing phosphorus [16]. Vanillin is one of the known natural compounds with antimicrobial properties [17]. It was determined that the polymeric films of vanillin derivatives also showed antimicrobial activity, which allow the use of vanillin-based polymers in food packing and medicine [18]. 

**Figure 1:**
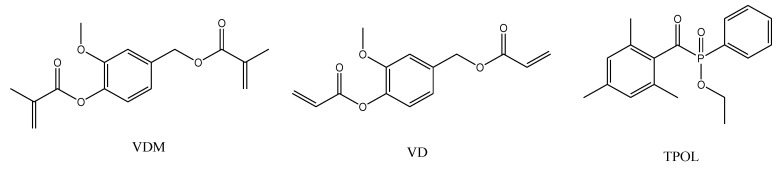
Chemical structures of vanillin dimethacrylate (VDM), vanillin diacrylate (VD), and ethyl(2,4,6-trimethylbenzoyl)phenylphosphinate (TPOL).

In this study, two commercially available vanillin derivatives, vanillin dimethacrylate (VDM) and vanillin diacrylate (VD), were tested in photocurable systems using ethyl(2,4,6-trimethylbenzoyl)phenylphosphinate (TPOL) as photoinitiator (Figure 1). TPOL was selected due to its high reaction rate and liquid form at room temperature [12]. TPOL is commonly used in optical 3D printing due to the photobleaching effect, which provides transparent coatings and allows coatings to cure in its lower layers [12]. The influence of the selected vanillin derivative, amount of photoinitiator, and the solvent’s to photocross-linking rate and rigidity of resulting polymers were evaluated by real-time photorheometry. The yield of insoluble fraction, cross-linking density, and swelling value of selected polymers were calculated to confirm the cross-linked structure. Antimicrobial activity of the selected polymer film was determined and compared with that of chitosan and oxyethyl starch films. Thermal and mechanical properties were investigated and compared with those of other acrylate polymers based on natural phenolics, acrylated epoxidized soybean oil-based polymers, and commercial petroleum-derived materials used in optical 3D printing.

## 2. Materials and Methods 

### 2.1. Materials

Vanillin dimethacrylate (VDM) and vanillin diacrylate (VD) (both from Specific Polymers), ethyl(2,4,6-trimethylbenzoyl)phenylphosphinate (TPOL, Fluorochem), dichloromethane (DCM, Reachem Slovakia), and chitosan and hydroxyethyl starch (both from Sigma-Aldrich) were used as received. The Autodesk Standard Clear Prototyping Resin (PR48) was received from Autodesk. FormLabs Clear FL6PCL02 resin was received from FormLabs.

### 2.2. Real-Time Photorheometry

UV/Vis curing tests were performed with resins containing 1 mol of vanillin derivative (VDM or VD) and 1, 3, or 5 mol.% of photoinitiator (TPOL) (Table 1) on a MCR302 rheometer (Anton Paar, Graz, Austria) equipped with the plate/plate measuring system. A minimal amount of DCM was used in some resins to dissolve solid components or for comparative investigations. The Peltier-controlled temperature chamber with the glass plate (diameter 38 mm) and the top plate PP08 (diameter 8 mm) was used. The measuring gap was set to 0.1 mm. The samples were irradiated by UV/Vis light in a wavelength range of 250–450 nm through the glass plate of the temperature chamber using UV/Vis spot curing system OmniCure S2000 (Lumen Dynamics Group Inc., Mississauga, ON, Canada). The temperature was 24 °C. Shear mode with the frequency of 10 Hz and shear strain of 0.9% were used in all cases. Storage modulus *G’*, loss modulus *G”*, loss factor tan*δ* (tan*δ* = *G”*/*G’*), and complex viscosity ŋ* were recorded as a function of irradiation time. *G’*, *G”*, and ŋ* values were taken after 350 s of UV/Vis irradiation (Table 2). The gel point *t_gel_* was defined as a crossover point of *G’* and *G”* modulus.

### 2.3. Preparation of Cross-Linked Polymer Specimens

The mixtures containing 1 mol of vanillin derivative (VDM or VD), 1, 3, or 5 mol.% of TPOL, and, if needed, a minimal amount of DCM (0.25 mL of DCM was used for 1 g of acrylate) were stirred at room temperature (25 °C) with a magnetic stirrer until homogenous phase was reached, then poured into a Teflon mold and cured for 1–4 min under a UV lamp (Helios Italquartz, model GR.E 500 W, Milan, Italy) with UV/Vis light at intensity of 310 mW/cm^2^.

### 2.4. Characterization Techniques

Fourier transform infrared spectroscopy (FT-IR) spectra were recorded using a Spectrum BX II FT-IR spectrometer (Perkin Elmer, Llantrisant, UK). The reflection was measured during the test. The range of wavenumbers was (650–4000) cm^−1^.

Cross-linked polymers **C3**–**C6** FT-IR (cm^−1^): 1714–1758, 1710–1732, 1730–1754 (ν, C=O), 1603, 1603–1649, 1603–1647 (ν, C=C), 737, 801–802 (ν, CH_2_).

The yield of insoluble fraction was determined by Soxhlet extraction. Polymer samples of 0.5 g were extracted with acetone for 24 h. After the extraction, the insoluble fractions were dried under vacuum until no changes of the weight were observed. The yield of insoluble fraction was calculated as a difference of the weight before extraction and after extraction and drying. 

Differential scanning calorimetry (DSC) measurements were performed on a DSC 8500 apparatus (Perkin Elmer, Llantrisant, UK). The heating rate of 20 °C/min under nitrogen atmosphere (50 mL/min) was used. The temperature ranged from −30 to 140 °C. A heating–cooling–heating cycle was used. Aluminum hermetic pans were used. The data were taken from the second heating curve.

Thermogravimetrical analysis (TGA) was performed on a TGA 4000 apparatus (Perkin Elmer, Llantrisant, UK). A heating rate of 20 °C/min under nitrogen atmosphere (100 mL/min) was chosen, and the temperature ranged from 10 to 800 °C. Aluminum oxide pans were used.

The top pressure test was performed on a BDO-FBO.5TH material testing machine (Zwick/Roell, Kennesaw, GA, USA). In all cases, the compression force was 5 N. The speed of compression was 50 mm/min. All tests were performed at 20 °C. The specimen was pressed with a cylindrical steel rod with a flat end of 8 mm diameter. The width of specimens was 30.0 (±0.00) mm and the thickness of specimens was 2.2 (±0.2) mm. In order to avoid the expansion of specimens to the sides during the test, the specimens were placed in a Teflon mold of the same size. To obtain accurate results, 10 tests for each specimen were made. The compression modulus was calculated by the following equation:

(1) $$E_{c}=\frac{F\cdot l_{0}}{S\cdot \Delta l}$$

where *E_c_* is a compression modulus (MPa); *S* is a surface area of specimen (mm^2^); *F* is a force (N); ∆*l* is the difference of an initial thickness of specimen and thickness of a loaded specimen (mm); and *l_0_* is an initial thickness of specimen (mm).

The bending test was performed on RSA-G2 Solids Analyzer (TA Instruments, New Castle, Delaware, USA). The thickness of specimens was 1.7 (±0.1) mm, the width was 5.00 (±0.00) mm, and the length was 40.00 (±0.00) mm. Tests were carried out at 20 °C. The speed of bending was 0.1 mm/s. Three-point contact bending geometry with a 25 mm gap between end contacts was used.

Bending modulus was calculated using the following equation:

(2) $$\delta =\frac{3\cdot F\cdot L}{2\cdot w\cdot d^{2}}$$

where *δ* is the bending modulus (Pa); *F* is the maximum force applied to the specimen (N); *L* is the length of the specimen (m); *w* is the width of the specimen (m); and *d* is the thickness of the specimen (m) [19].

The swelling value of the cross-linked polymer specimens was obtained by measuring the volume of specimens swollen in chloroform and toluene at 25 °C. The polymer film specimens of 15 (±0.1) mm length, 5 (±0.00) mm width, and 0.5 (±0.05) mm thickness were used. A glassy container comprised two spherical parts of 50 mL joined by a tube graduated with the accuracy of 0.02 mL was used. One spherical part was separated from the graduated tube by a liquid-permeable partition. This spherical part was fitted with the neck closed by a stopper. The swelling agent was poured, and the sample was placed in through this neck. The initial volume of the polymer film was measured before placing into the measuring container. The measuring container was kept in such position that a sample was immersed in the swelling agent during the test and turned in such position that the liquid leaked to another side of the container at every 5 min. The change of the volume of the swelling agent was measured. The swelling value was calculated by the following equation:

(3) $$\alpha =\frac{V-V_{0}}{V_{0}}\cdot 100$$

where *α* is a swelling value (%); *V* is a volume of swollen specimen (mL); and *V_0_* is an initial volume of specimen (mL).

The antimicrobial activity of the polymers was estimated quantitatively by inoculating specimens with microbial suspension. The test microorganisms were Gram-negative bacterium *Escherichia coli* ATCC 25,922 (*E. coli*) and Gram-positive bacterium *Staphylococcus aureus* ATCC 29,213 (*S. aureus*). The concentration of inoculum suspension was assessed with a spectrophotometer (Evolution 60S, Thermo Fisher Scientific, Waltham, MA, USA) and then corrected by seeding the suspension on Mueller Hinton agar (MHA). The final inoculum concentration of *E. coli* was 7 × 10^5^ and 8 × 10^5^ CFU/mL of *S. aureus*. The films of chitosan and hydroxyethyl starch prepared by casting from aqueous solutions were used for comparison. The testing specimens of **C4**, chitosan, and hydroxyethyl starch in dimensions of 10 × 10 mm were placed into sterile Petri dishes of 50 mm diameter, inoculated with 10 µL of prepared bacterial suspension, and incubated in humid chambers at (35 ± 2) °C. After 2, 6, and 24 h the specimens were washed with 2 mL of sterile 0.85% saline, and serial dilutions of culture suspensions were sown on MHA in Petri dishes. The dishes with bacteria were incubated for 48 h at (35 ± 2) °C. After incubation, colony numbers were counted, and percent reduction was calculated by the following equation: (*a* − *b*)/*a* × 100%, where *a* is a concentration of colony forming units (CFU/mL) in inoculum; and *b* is a mean of recovered bacteria (CFU/mL) on specimens from triplicate after incubation.

### 2.5. Optical Micro-Fabrication Techniques

**Figure 2:**
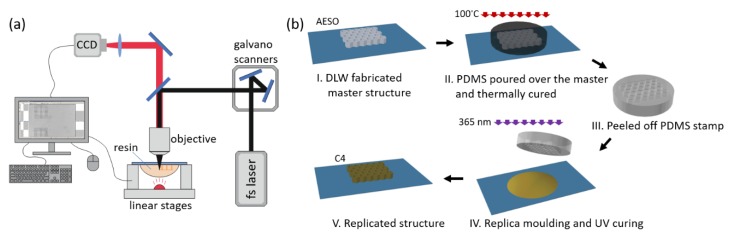
(**a**) Optical setup for direct laser writing (DLW) 3D lithography. (**b**) Steps of making the replica by the microtransfer molding (μTM) technique.

Two types of optical printing techniques were used to produce 3D objects out of custom made photocross-linkable resins. At first, direct laser writing (DLW) 3D lithography experiments were conducted employing a Pharos laser (515 nm, 300 fs, 200 kHz, Light Conversion Ltd., Vilnius, Lithuania), 20 × NA = 0.8 and 63 × NA = 1.4 objectives, and combined movement of the linear stages and galvano-scanners. The setup is shown in Figure 2a, and its detailed description can be found in a previous publication [20]. The goal was to figure out if the resins were suitable for ultrafast laser pulse initiated 3D polymerization. Woodpile structures were chosen as test objects. Their 3D model consisted of two layers of 2D gratings, connected by vertical columns 20 μm high. The gratings comprised orthogonal sets of logs 15 μm wide and 75 μm long with a 15 μm gap between them, resulting in a 30 μm period of the structure. Each log was fabricated by performing multiple parallel scans, and the number depended on the distance *d_xy_* between them. *d_xy_* was set to 0.25 and 0.5 μm. Optimal exposure parameters were determined by varying the laser power (*P*), which corresponded to the light intensity (*I*) at the sample and scanning velocity (*v*). During the fabrication, the resin was placed on a glass substrate through which it was irradiated by the laser beam. After the exposure, the samples were developed in dichloromethane for 30 min, removing the uncured resin and leaving only the produced structures on the substrate.

Secondly, a microtransfer molding (μTM) technique, also known as nanoimprint lithography, was employed [21]. Steps involved in making the replica are depicted in Figure 2b. First, a master structure was fabricated out of acrylated epoxidized soybean oil (AESO) by DLW. Polydimethylsiloxane (PDMS) was poured over this structure and thermally cured at 100 °C for 1 h, thus creating a soft mold (stamp). It was then used to make a replica out of **C4** resin. The chosen structure was a 3D sculpture of Marvin—a SLT model, used as a benchmark sample for the material testing in the 3D printing community. A UV diode emitting 365 nm wavelength light (CS2010, Thorlabs, Newton, NJ, USA) was used to cure the **C4** resin and obtain the replica.

The fabricated structures were characterized using scanning electron microscopes (SEM, Hitachi TM-1000, Tokyo, Japan and Prisma E, Eindhoven, The Netherlands). 

## 3. Results

### 3.1. Monitoring of Photocross-Linking Kinetics by Real-Time Photorheometry

**Figure 3:**
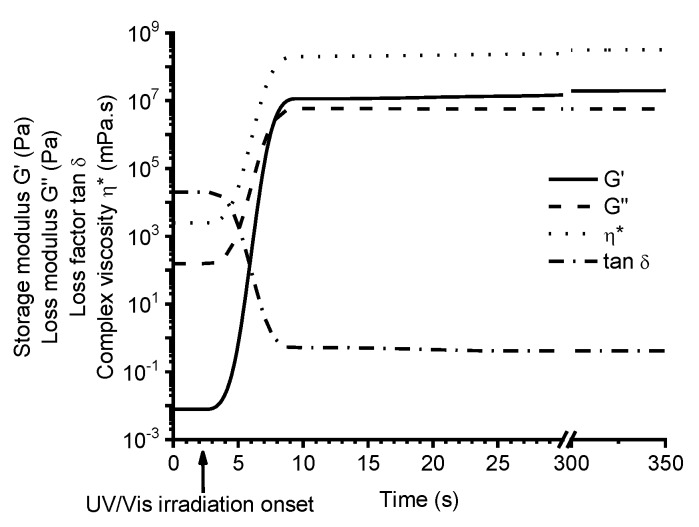
Dependencies of storage modulus *G’*, loss modulus *G”*, loss factor tan*δ*, and complex viscosity η* of resin **C9** on irradiation time, at 24 °C.

The photopolymerization of vanillin acrylate-based (VD and VDM) resins with various concentrations of TPOL as photoinitiator was studied by real-time photorheometry. As an example, Figure 3 shows the evolution of storage modulus *G’*, loss modulus *G”*, loss factor tan*δ*, and complex viscosity η* of the VDM-based resin **C9** during UV/Vis irradiation. The cross-linking process began when the values of *G’*, *G”*, and η* started to increase. The gel point (*t_gel_*) (defined as *G’*=*G”*) [22] of resin **C9** was reached after 6 s from UV/Vis irradiation onset. As irradation of the resin proceeded with time, the values of *G’*, *G”* modulus, and η* continued to increase due to gel aging and settling down into a steady-state, indicating the end of the cross-linking process. All vanillin acrylate-based resins showed similar behaviours. Real-time photorheometry data of all resins are summarized in Table 2.

**Figure 4:**
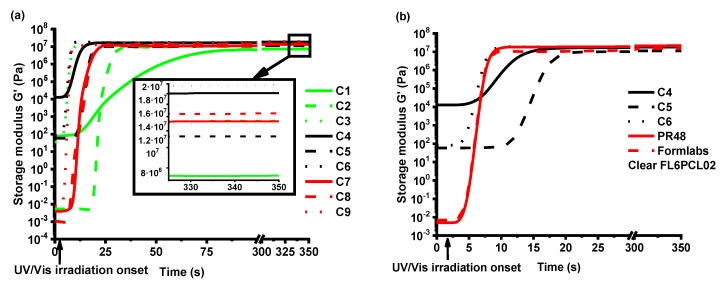
(**a**) Dependencies of storage modulus *G’* of the resins **C1**–**C9** on irradiation time. Concentration of TPOL in the resins: green curves—1 mol.%, black curves—3 mol.%, red curves—5 mol.%. (**b**) Dependencies of storage modulus *G’* of the resins **C4**–**C6**, FormLabs Clear FL6PCL02 and PR48 on irradiation time, at 24 °C.

As the storage modulus *G’* characterizes the rigidity of the formed thermosetting polymers [23], the dependencies of *G’* on irradiation time of the resins with 1, 3, and 5 mol.% of TPOL are shown in Figure 4a. Comparing the shape of the *G’* curves of the resins with different concentrations of photoinitiator, the fastest photocross-linking and the highest final rigidity were demonstrated by resin **C4** with 3 mol.% of TPOL in the VD-based resins without solvent series (**C1**, **C4,** and **C7**). The presence of solvent had a higher influence on the photocross-linking rate than the concentration of photoinitiator. In VD-based resins with solvent series (**C2**, **C5,** and **C8**), the photocross-linking rates of two resins with 3 mol.% and 5 mol.% of TPOL (**C5** and **C8** respectively) were very similar and faster than that of resin **C2**, although a more rigid polymer was obtained from resin **C8**. In VDM-based resins with solvent series (**C3**, **C6,** and **C9**), the photocross-linking rate did not depend on the concentration of TPOL, although a more rigid polymer was obtained from resin **C9** with 5 mol.% of TPOL. Comparing the gel points (*t_gel_*) of the resins (Table 2), the *t_gel_* was reached the fastest when 3 mol.% of the TPOL was used in the case of all resin series. When a higher amount of photoinitiator was used, the effectiveness of photocross-linking was reduced due to the rapid photocross-linking of the surface layer, which reduced light penetration into the deeper layers of the material [24]. The addition of the solvent into the resin slowed down the photocross-linking process, and less-rigid polymers were obtained. For example, the resin **C4** without solvent reached the gel point after 6 s and a rigidity of 18.10 MPa was obtained, while resin **C5** reached the gel point only after 12 s and obtained a rigidity of 11.30 MPa (Table 2). This could be due to the solvent action as a chain transfer agent which slows down the photocross-linking process [25]. The photocross-linking of all VDM-based resins (**C3**, **C6,** and **C9**) was faster compared to all VD-based resins (**C1**, **C2**, **C4**, **C5**, **C7,** and **C8**), although the acrylate group was earlier described as more active than the methacrylate group [26]. This could be due to the darker color of VD-based resins compared with VDM-based resins that could cause a slower polymerization process by making it harder for the light to reach and cure the deeper layers of the resins [27].

The series of resins with 3 mol.% of TPOL (**C4**, **C5,** and **C6**) were selected for further investigations according to the photocross-linking rate and polymer rigidity. These resins were compared with commercial acrylate resins for optical 3D printing, FormLabs Clear FL6PCL02 and PR48 (Figure 4b). Both commercial resins demonstrated similar photocross-linking rates (both *t_gel_* = 6 s) as resin **C4** (*t_gel_* = 6 s) (Table 2). However, resin **C6** demonstrated a slightly higher photocross-linking rate (*t_gel_* = 5 s) than commercial resins. Moreover, the rigidity of resins **C4** (*G’* = 18.10 MPa) and **C6** (*G’* = 18.20 MPa) was slightly higher than that of FormLabs Clear FL6PCL02 (*G’* = 15.20 MPa), but less than that of PR48 (*G’* = 21.40 MPa). Resin **C5** demonstrated poor behavior compared with commercial resins. The photocross-linking was slower (*t_gel_* = 12 s) and the rigidity was the lowest (*G’* = 18.10 MPa) from all the selected resins **C4**, **C6**, and commercial resins (Table 2).

### 3.2. Characterization of Photocross-Linked Polymer Structure

**Figure 5:**
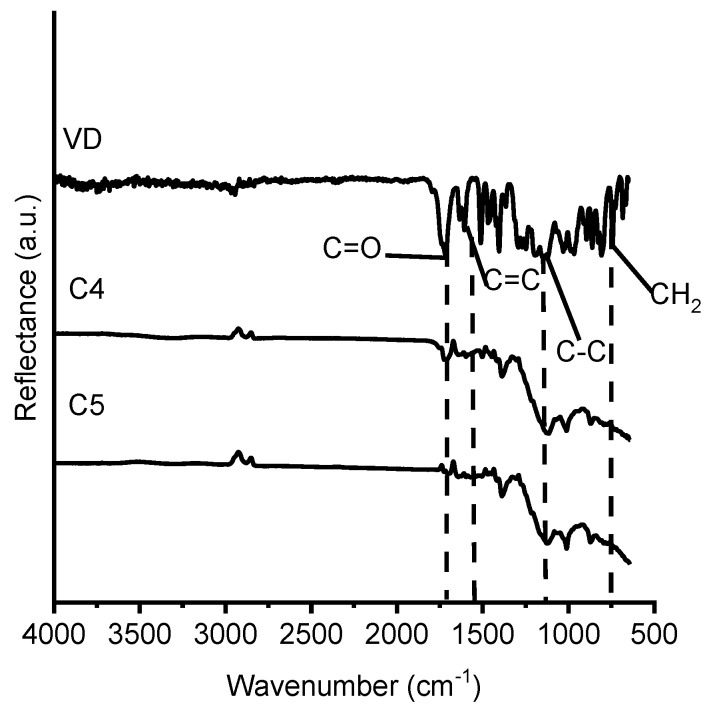
FT-IR spectra of VD and the cross-linked polymers **C4** and **C5.**

The chemical structure of the vanillin-based polymers was identified by FT-IR spectroscopy. The signals of C=C group, which were present at 1607 cm^−1^, and C=O group, which were present at 1730 cm^−1^, in the FT-IR spectra of VDM and VD were reduced in the polymer spectra. The large increase of C-C group signal was detected at 1128–1131 cm^−1^, which shows the formation of polymer. As an example, the FT-IR spectra of VD and the cross-linked polymers **C4** and **C5** are presented in Figure 5. 

**Figure 6:**
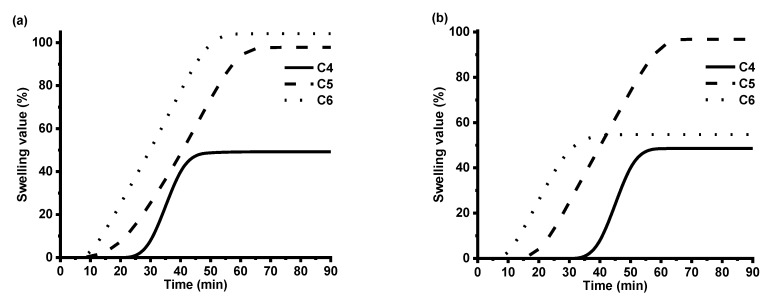
The swelling values of polymers **C4**–**C6** in chloroform (**a**) and toluene solvent (**b**)

To confirm the formed cross-linked structure of polymers, Soxhlet extraction was performed. The highest value of the yield of insoluble fraction was obtained for polymer **C4,** for which synthesis of VD was used without addition of DCM in comparison to polymer **C5** (96% in comparison to 77%) (Table 3). Swelling tests are of great importance to characterize the network structure. The higher swelling values showed that longer chains between the cross-linking points were formed in the polymer. Both C4 and C5 are vanillin diacrylate-based polymers; the only difference is that a small amount of DCM was used in the reaction mixture of C5, which resulted in the formation of the lower yield of insoluble fraction (Table 3) and the lower crosslinking density confirmed by the higher swelling values of polymer C5 (Figure 6, Table 3). C6 is vanillin dimethacrylate-based polymer in which preparation of a small amount of DCM was used; thus, higher swelling values were obtained in chloroform. Some other factors such as the polymer–solvent interaction can affect swelling properties as well. Vanillin dimethacrylate is soluble in chloroform and insoluble in toluene. The poor interaction of vanillin dimethacrylate-based polymer chains with toluene was the reason why the swelling of polymer C6 in toluene was worse than that in chloroform (Figure 6).

### 3.3. Thermal Properties of Photocross-Linked Polymers

**Figure 7:**
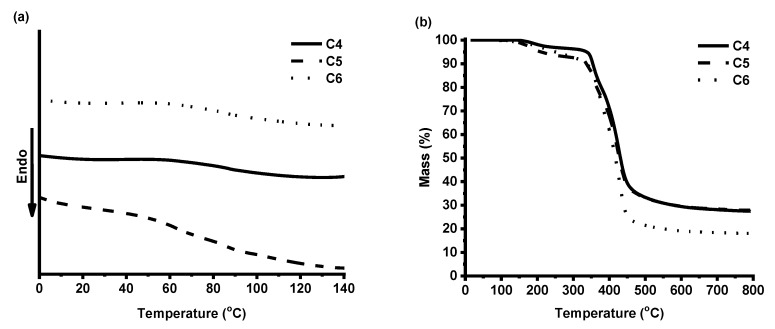
DSC thermograms (**a**) and thermogravimetric curves (**b**) of cross-linked polymers **C4**–**C6.**

DSC and TGA were used to study the thermal characteristics of the photocross-linked polymers **C4**–**C6**. Synthesized polymers are amorphous materials; therefore, only a glass transition was obtained in DSC curves (Figure 7a). The glass transition temperatures (T_g_) of polymers **C4** and **C6** were very similar (87 and 86 °C, respectively) and depended on the cross-linking density and the yield of insoluble fraction. A lower glass transition temperature was observed for polymer **C5** (63 °C), with the lowest cross-linking density determined from the swelling test results. The glass transition temperatures of obtained polymers were similar to the acrylate resins based on natural phenolics, presented as candidate materials for stereolithography (79 °C) [28].

Thermal decomposition of the synthesized polymers occurred in two steps (Figure 7b). The first step in the TGA curves can be explained by the presence of the branched or linear macromolecules. The temperature of 10% weight loss (*T_dec.-10%_*) of the VD-based polymer **C4** (350 °C) was higher than that of the VD-based polymer **C5** prepared using DCM (330 °C) and the VDM-based polymer **C6** (340 °C). Higher *T_dec.-10%_* correlated with the higher yield of insoluble fraction and cross-linking density. The temperature of 10% weight loss of obtained polymers was similar or even slightly higher than that of some acrylated epoxidized soybean oil-based polymers (297–356 °C) tested in 3D printing [20].

### 3.4. Mechanical Properties of Photocross-Linked Polymers

Only resin **C4** was suitable for the preparation of the specimens for bending and top pressure tests because the specimens of resins **C5** and **C6** cracked during the photocross-linking process, and it was impossible to make uniform specimens for these tests. To improve the mechanical properties of these polymers, addition of reactive diluents will be carried out in further studies. Commercial photoresins PR48 and FormLabs Clear FL6PCL02 were tested in the same conditions to compare their mechanical characteristics with those of polymer **C4**. The mechanical testing results are summarized in Table 4. All polymers demonstrated low deformation during compression tests. Polymer **C4** demonstrated a lower compression modulus than that of the polymers prepared from the commercial resins PR48 and FormLabs Clear FL6PCL02, but a much higher compression modulus than the acrylated epoxidized soybean oil based polymers (0.19–0.66 Pa) tested in 3D printing [20]. The specimens of all polymers did not break during the bending test. Very similar 30% specimen bending forces and bending modulus were obtained for polymers **C4** and PR48. The specimen prepared from FormLabs Clear FL6PCL02 demonstrated a lower 30% specimen bending force and bending modulus.

According to the results, resin **C4** was selected for further testing by optical micro-fabrication techniques, and the testing of antimicrobial activity was carried out.

### 3.5. Antimicrobial Activity

**Figure 8:**
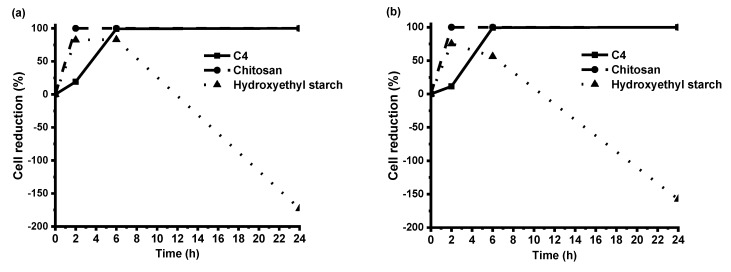
The reduction of *E. coli* (**a**) and *S. aureus* (**b**) cells during 24 h of contact time with specimens of polymer **C4,** chitosan, and hydroxyethyl starch.

The testing of antimicrobial activity was performed for polymer **C4** and two reference polymer films of chitosan and hydroxyethyl starch. The results of antibacterial activity testing demonstrated that polymer **C4** killed 99% of both Gram-negative *E. coli* and Gram-positive *S. aureus* after 6 h of contact time, and after 24 h, all bacteria on this film were lifeless (Figure 8). Bacteria on the hydroxyethyl starch film, having no antibacterial activity, even began to propagate after 2 contact hours. In comparison with chitosan, which is known to have a very strong antibacterial activity [29], polymer **C4** showed only a bit weaker activity. In our experiment, bacteria on the chitosan film were killed completely after 2 contact hours. These results are in agreement with other studies indicating significant antimicrobial effects of vanillin and vanillin incorporation into complex compounds [18,30,31].

### 3.6. Characterization of Optically Printed Structures

**Figure 9:**
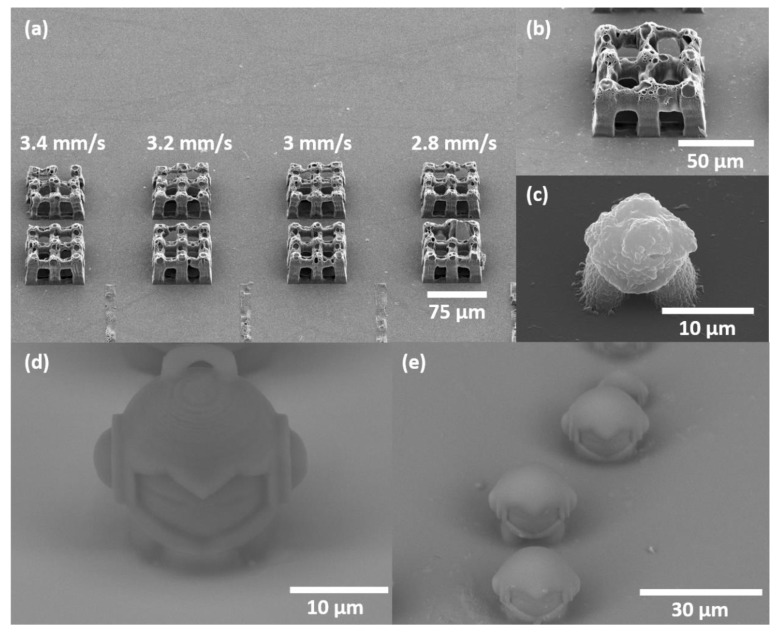
(**a**) Array of the 75 × 75 µm^2^ size woodpile structures out of **C4** resin: *v* varies from 2.8 to 3.4 mm/s, *P* = 0.4 mW (*I* = 1.2 TW/cm^2^) and *d_xy_* = 0.25 μm (lower row), *P* = 0.5 mW (*I* = 1.5 TW/cm^2^) and *d_xy_* = 0.5 μm (upper row). (**b**) Close-up of the 75 × 75 µm^2^ woodpile structure: *v* = 1 mm/s, *P* = 0.3 mW (*I* = 0.9 TW/cm^2^), *d_xy_* = 0.25 μm. (**c**) Sculpture of Marvin out of **C4** resin: *v* = 1.8 mm/s, *P* = 0.1 mW (*I* = 0.3 TW/cm^2^), *d_xy_* = 0.25 μm. (**d**) Sculpture of Marvin out of AESO: *v* = 1.2 mm/s, *P* = 0.18 mW (*I* = 0.6 TW/cm^2^), *d_xy_* = 0.25 μm. (**e**) Sculptures of Marvin out of **C4** resin molded from the master structure (section (**d**)) via microtransfer molding; 365 nm wavelength light source was used for curing. Objects in sections (**a**) and (**b**) were manufactured with 20 × 0.8 NA objective, (**c**) and (**d**) 63 × 1.4 NA. All SEM images were obtained at 45° angle.

A test to assess the optimal fabrication parameters was performed, and the capability to produce 3D microporous woodpile structures out of the resin **C4** via DLW was demonstrated. An array of 75 × 75 μm^2^ woodpiles is shown in Figure 9a,b. Manufacturing parameters and focusing conditions are provided in the caption of Figure 9.

Produced objects corresponded to the used 3D model, however with deviations including tilted columns and not fully formed logs. These could happen due to the too low degree of cross-linking, which causes shrinkage, when *d_xy_* 0.5 μm was applied. Also, the voids on the woodpile’s surfaces were observed. The voids were caused by bubbles, which have appeared due to the inhomogeneity of the resin, resulting in the deviation of the optical damage threshold through the whole material. Thus, in exposing resin **C4** with tightly focused laser irradiation, some parts were over-exposed and induced bubble formation. Using a higher numerical aperture objective, a sculpture of Marvin was produced (Figure 9c). The basic shape of the given STL model was polymerized, but detailed parts of the face, ears, and the loop were missing. To obtain a well-defined Marvin out of **C4** resin, a μTM technique was employed. A master structure of Marvin was produced out of AESO (Figure 9d). The replicated structures of Marvin were manufactured by curing the **C4** resin inside a PDMS mold with UV diode. The obtained objects are demonstrated in Figure 9e. In this case, the produced Marvin had a smooth surface and detailed parts, except loops, which were not replicated due to the limited capability of the μTM technique to replicate closed loops. Differences in material surface appearance emerged because of the different illumination conditions. Using DLW, objects were made of stitched scanning point-by-point manner and thresholded light-matter interaction, sensitive to the material’s inhomogeneity. On the other hand, with μTM a uniform UV irradiation distribution resulted in homogeneous curing. 

## 4. Conclusions 

Novel vanillin acrylate-based resins were designed and investigated as candidate materials for optical 3D printing. The kinetics of photocross-linking of vanillin dimethacrylate or vanillin diacrylate using ethyl(2,4,6-trimethylbenzoyl)phenylphosphinate as photoinitiator were investigated by real-time photorheometry. The photocross-linking of the vanillin dimethacrylate-based resins was faster compared with the vanillin diacrylate-based resins. The gel point was reached the fastest when 3 mol.% of photoinitiator was used. The addition of solvent into the resin slowed down the photocross-linking process, and less-rigid polymers were obtained. The vanillin diacrylate-based polymer prepared without solvent possessed higher values of yield of insoluble fraction, thermal stability, and lower swelling values in comparison to the vanillin dimethacrylate-based polymer. Only the vanillin diacrylate-based polymer prepared without solvent was appropriate for testing of mechanical properties. It demonstrated similar properties to those of the commercially available photocurable resins. Subsequently, the possibility to optically structure this resin was successfully demonstrated. Although acquisition of a smooth surface of the structures proved to be challenging when employing DLW 3D lithography due to material inhomogeneity, the microtransfer molding technique allowed to overcome this limitation. For the improvement of the mechanical properties of other polymers, the addition of reactive diluents will be carried out in further studies. Vanillin diacrylate polymer film showed a significant antimicrobial effect only a bit weaker than that of chitosan film.

## Figures and Tables

**Table 1:** Composition of the resins **C1**–**C9**.

Resin	Vanillin Derivative	Solvent	Amount of Photoinitiator TPOL, mol.%
**C1**	VD	-	1
**C2**	VD	DCM	1
**C3**	VDM	DCM	1
**C4**	VD	-	3
**C5**	VD	DCM	3
**C6**	VDM	DCM	3
**C7**	VD	-	5
**C8**	VD	DCM	5
**C9**	VDM	DCM	5

**Table 2:** Rheological characteristics of the resins **C1**–**C9.**

Resin	Storage Modulus *G’*, MPa	Loss Modulus *G”*, MPa	Complex Viscosity η*, MPa·s	Gel Point ^a^ *t_gel_*, s
**C1**	7.35	6.34	0.15	14
**C2**	13.40	2.35	0.22	20
**C3**	13.00	1.65	0.21	6
**C4**	18.10	2.70	0.29	6
**C5**	11.30	1.64	0.18	12
**C6**	18.20	2.94	0.29	5
**C7**	13.30	5.78	0.23	10
**C8**	14.50	2.02	0.23	14
**C9**	19.80	3.36	0.32	6
**FormLabs Clear FL6PCL02**	15.20	3.35	0.25	6
**PR48**	21.40	4.19	0.35	6

^a^ calculated from the UV/Vis irradiation onset.

**Table 3:** Characteristics of the cross-linked vanillin-based polymers.

Polymer	Yield of Insoluble Fraction, %	Swelling Value in Chloroform, %	Swelling Value in Toluene, %
**C4**	96	49.22 ± 0.06	48.62 ± 0.06
**C5**	77	97.83 ± 0.06	96.85 ± 0.06
**C6**	89	104.21 ± 0.06	54.72 ± 0.06

**Table 4:** Mechanical characteristics of the cross-linked vanillin-based polymers.

Polymer	Deformation during Compression, %	Compression Modulus, MPa	Force of Specimen Bend of 30 %, N	Bending Modulus, MPa
**C4**	4.95 ± 0.02	2.01 ± 0.02	3.65 ± 0.04	9.47 ± 0.04
**PR48**	1.79 ± 0.02	5.56 ± 0.02	3.39 ± 0.07	8.80 ± 0.07
**FormLabs Clear FL6PCL02**	1.39 ± 0.02	7.17 ± 0.02	0.42 ± 0.02	1.09 ± 0.02
